# Ocean acidification alters early successional coral reef communities and their rates of community metabolism

**DOI:** 10.1371/journal.pone.0197130

**Published:** 2018-05-30

**Authors:** Sam H. C. Noonan, Anna Kluibenschedl, Katharina E. Fabricius

**Affiliations:** 1 Australian Institute of Marine Science, Townsville, Queensland, Australia; 2 Department of Marine Science, University of Otago, Dunedin, New Zealand; Department of Agriculture and Water Resources, AUSTRALIA

## Abstract

Ocean acidification is expected to alter community composition on coral reefs, but its effects on reef community metabolism are poorly understood. Here we document how early successional benthic coral reef communities change *in situ* along gradients of carbon dioxide (CO_2_), and the consequences of these changes on rates of community photosynthesis, respiration, and light and dark calcification. Ninety standardised benthic communities were grown on PVC tiles deployed at two shallow-water volcanic CO_2_ seeps and two adjacent control sites in Papua New Guinea. Along the CO_2_ gradient, both the upward facing phototrophic and the downward facing cryptic communities changed in their composition. Under ambient CO_2_, both communities were dominated by calcifying algae, but with increasing CO_2_ they were gradually replaced by non-calcifying algae (predominantly green filamentous algae, cyanobacteria and macroalgae, which increased from ~30% to ~80% cover). Responses were weaker in the invertebrate communities, however ascidians and tube-forming polychaetes declined with increasing CO_2_. Differences in the carbonate chemistry explained a far greater amount of change in communities than differences between the two reefs and successional changes from five to 13 months, suggesting community successions are established early and are under strong chemical control. As pH declined from 8.0 to 7.8, rates of gross photosynthesis and dark respiration of the 13-month old reef communities (upper and cryptic surfaces combined) significantly increased by 10% and 20%, respectively, in response to altered community composition. As a consequence, net production remained constant. Light and dark calcification rates both gradually declined by 20%, and low or negative daily net calcification rates were observed at an aragonite saturation state of <2.3. The study demonstrates that ocean acidification as predicted for the end of this century will strongly alter reef communities, and will significantly change rates of community metabolism.

## Introduction

The oceanic uptake of anthropogenic carbon dioxide (CO_2_) emissions is causing ocean acidification (OA) [1]. OA not only lowers seawater pH, but also reduces the saturation state (Ω) of calcium carbonate (CaCO_3_) minerals, and increases CO_2_ and bicarbonate ion concentration. Predicting how marine communities will respond to OA is complicated, as many of these chemical alterations can act as drivers of change [1,2]. For example, the inhibition of calcification from declining pH and Ω [3], or the stimulus of photosynthesis from the increases in dissolved inorganic carbon (C_T_) [2], may affect species performances. Such physiological responses may also cause disruptions of ecological interactions, further altering communities [4,5]. As OA is occurring progressively, the response of species and communities is likely to occur along a continuum as well. Individual species have displayed both linear responses [6,7], as well as non-linear thresholds or tipping points [8] along gradients of CO_2_, while the response of communities remains largely uninvestigated. To better predict how communities will be shaped under OA, there is thus a need for studies which investigate the response curves of communities to increasing CO_2_.

Coral reefs are likely to be among the ecosystems most affected by OA [9]. Predictions are based on a multitude of single-species physiological studies [10], and several that have investigated changes at the community level. Community scale studies have centred around naturally occurring high-CO_2_ analogues, such as volcanic CO_2_ seep sites [11–13] or other oceanographic features affecting their carbonate chemistry [14–16], as well as larger-scale multi-species tank experiments [17–19]. While there is substantial variation in the responses between taxa, the general consensus predicts declines in biodiversity, a retraction of many calcifying species (e.g. scleractinian corals, coralline algae and foraminifera), an expansion of non-calcifying phototrophs (e.g. algae and seagrasses), and increased bioerosion. [20].

Coupled with the predicted changes in community composition under OA will likely be changes in community metabolism. However, scaling up OA effects on metabolic processes from individuals and species to the community level has proven difficult, and our current understanding is poor [21]. To date the best inferences have been based on naturally occurring seasonal carbonate chemistry changes [22–24], or the manipulation of seawater carbonate chemistry on coral reefs *in situ* [25,26] and in experimentation [18,27], as well as larger-scale mesocosm experiments [17,19,28,29]. These studies generally predict that rates of community photosynthesis and respiration will remain relatively unchanged from the reefs of today, while calcification and net CaCO_3_ accumulation will decline. However, these investigations have mainly examined effects due to changes in seawater carbonate chemistry, without fully accounting for changes due to the longer-term shifts in benthic community composition that may occur under OA. For example, Ω declines may directly reduce calcification rates in numerous taxa [10], but if these taxa are then outcompeted by non-calcifers, community calcification rates may further decline. Similarly, OA can increase rates of community production by directly stimulating photosynthesis in some species [30,31], or indirectly by increasing the benthic cover of certain phototrophs [2]. To gain further insight into the community metabolic dynamics of coral reefs under OA, measurements must be made on communities that have developed in their entirety under altered seawater carbonate chemistries.

The frequency and severity of disturbances affecting coral reefs is increasing [32], and scleractinian coral cover is now often well below 30% [33]. Scleractinian corals eventually re-enter communities, however it is early-successional non-scleractinian taxa (e.g. algae, sponges, and other sessile invertebrates) that increasingly dominate light exposed benthic reef communities [34]. Furthermore, shade exposed cryptic taxa within crevices of the reef matrix can account for the largest fraction of biomass in reef systems [35]. Both of these communities—the early successional benthic taxa on illuminated and shaded surfaces—are often overlooked in reef community metabolism studies, although their metabolism co-determines the carbonate chemistry conditions for newly settling corals within the benthic boundary layer.

In this study we investigate how OA will shape the composition of early-successional benthic communities that live on the carbonate substrata of coral reefs, and the metabolic rates of the non-scleractinian components of reef communities that have developed under altered carbonate chemistries. To do so, non-carbonate settlement tiles were deployed *in situ*, under natural levels of light and shade, temperature and water flow, along CO_2_ gradients at two volcanic CO_2_ seep and two control sites in Papua New Guinea. Benthic communities developing on the upper light exposed tile sides, as well as the shaded crevice-dwelling taxa on the lower sides were investigated after five and 13 months, and their successional changes, and taxa-specific responses along the CO_2_ gradients were explored. Response curves in community photosynthesis, respiration and light and dark calcification were then determined after 13 months.

## Materials and methods

### Study site and carbonate chemistry

This study was conducted at two tropical shallow water (<5 m depth) CO_2_ seeps and adjacent control sites in Milne Bay Province, Papua New Guinea. Seep and control sites at Upa-Upasina and Dobu are located adjacent to Normanby and Dobu Islands, respectively, and are described in detail by Fabricius *et al*. [11]. At both seep sites there is an area where near-pure (~99%) CO_2_ gas emerges from the seafloor, locally altering the carbonate chemistry of the seawater without altering its temperature [31]. The seeping has resulted in an altered benthic community, with hard and soft coral diversity, calcifying algal cover and the abundance and diversity of numerous mobile invertebrate taxa declining, while non-calcifying algae and seagrass cover is increased [4,11]. This research was conducted under permits issued to Dr Katharina Fabricius from Papua New Guinea’s Department of Environment and Conservation, and the National Research Institute.

Data on the seawater carbonate chemistry for the sites has been published by Fabricius *et al*. [4,11], as have seawater measurements above settlement tiles used in this study [8,20,36]. Specifically, during four ~2-week expeditions between 2011 and 2013, 911 pH measurements were recorded across the 90 settlement tiles (median n = 8 per tile), and a further 625 samples (median n = 5 per tile) were taken for total alkalinity (A_T_) [8]. Given seeping intensity varies spatially and temporally within the mosaic of gas streams that comprises the seep sites, these samples were used to form long-term medians of the seawater carbonate chemistry at the exact location of each tile. Unless otherwise stated, all pH values are presented in the total scale (pH_T_).

### Benthic community composition

In December 2011, 90 labelled settlement tiles were evenly distributed across seep and control sites at both Dobu and Upa-Upasina reefs (n = 15 control and 30 seep site tiles per reef). Tiles were 11.5 * 11.5 cm, made of 3 mm thick polyvinyl chloride (PVC) and roughened on both sides with sandpaper. Kennedy *et al*. [37] have shown that the percent coverage of benthic taxa settling on PVC tiles more closely matched adjacent reef substrata than other tile materials. Tiles were deployed horizontally on numbered baseplates, at ~3 m depth, ~2 cm from the reef substratum, >2m apart. The gap between the tile and the reef substrate allowed communities to not only develop on the upper light exposed tile side, but also for cryptic taxa to recruit to the lower shaded side. The tiles were first collected after five months (May 2012), photographed on both sides while being continuously submerged, and redeployed to their original location. In January 2013 (after 13 months deployment) the tiles were again collected, physiological measurements were made (details below), and they were rephotographed, before being dried and transported to the laboratories at the Australian Institute of Marine Science. Eighty-eight of the 90 settlement tiles were recovered during both census periods, with one missing from each seep site at Dobu and Upa-Upasina.

The benthic community composition of the upper and lower sides of the tiles, from the two census periods, was assessed on the tile photographs using the evenly spaced point analysis method [38]. To do so, photographs were imported into image editing software (Photoshop CS6, Adobe Systems, USA), and digitally overlaid with a grid consisting of 7 * 7 evenly spaced lines. The identity of the benthos occurring under the resultant 49 cross points was recorded and classified into 15 operational taxonomic units (OTUs, S1 Table), including seven groups of algae and cyanobacteria, six phyla of benthic invertebrates, bare tile space, and a category for any taxa that could not be identified. The point counts were converted into percent coverage data and some OTUs were further grouped into functional categories (non-calcifying algae, calcifying and non-calcifying invertebrates) for later statistical analysis (S1 Table). Photographs from 15 of the 30 seep tiles at Upa-Upasina at the 13 month census were lost (the camera memory card was accidently formatted prior to backup) and hence not included in analyses. The abundances of sessile tube-forming polychaetes and of coral recruits were counted directly on each tile once dried in the laboratory using a dissection microscope.

### Benthic community metabolism

To determine rates of community gross photosynthesis, dark respiration, and light and dark calcification, all settlement tiles were incubated in the light and dark on the day of their collection in January 2013. For the incubations, tiles were transferred from their holding containers in running seawater into clear rectangular-prism chambers (920 mL volume), atop spacers which left ~1.5 cm gap between the tile and the walls, bottom and lid of the chamber. Chambers were placed in black flow-through bins that served as water baths and were maintained at ambient *in situ* temperatures from a 2m deep intake (30°C). To minimise boundary layers, a 35 mm magnetic stirrer bar was placed into each chamber, activated with a custom made system of rotating magnets and pullies placed under the water baths. Communities from control sites were incubated in seawater at pH_T_ 8.08, while those originating from the seep sites were placed in seawater with pH_T_ 7.70. Four chambers per seep, and two per control site, were incubated with a blank settlement tile as a control. Water obtained from the seep sites was mixed with control seawater immediately prior to incubations to make a large batch at the target pH level, which was then used to fill the chambers. pH was determined with a portable pH meter (SG23, Mettler Toledo, USA) calibrated on the NBS scale. Measurements of pH_NBS_ and A_T_ from the incubation water were used to calculate carbonate chemistry parameters using the macro CO2SYS with the constraints set by Dickson and Millero [39], following Lewis *et al*. [40] (S2 Table).

Light incubations were conducted in black bins under four white fluorescent tubes (10 000K) at their maximum output of 180 μmol photons m^-2^ s^-1^. After ~90 minutes, the O_2_ concentration in each chamber was measured (meter: HQ30d; probe: LDO101 IntelliCAL, Hach, USA) and a sample of water was retained and fixed with saturated HgCl_2_ (>7 g L^-1^) for calcification assays. Tile communities were allowed >30 min dark adaptation in the water baths under black lids, before the chambers were closed again and the process was repeated in the dark. Light and dark calcification rates were determined with the alkalinity anomaly technique [41], using open cell titration (Metrohm 855 robotic titrosampler, Switzerland) fitted with a gran function following Vogel *et al*. [42]. The alkalinity anomaly technique assumes incubation water A_T_ is only affected by the removal (calcification) or addition (dissolution) of CaCO_3_, however some organisms in the tile communities (e.g. molluscs) would have altered A_T_ through the release or uptake of nutrients [43]. This would have added some unaccounted measurement error to calcification rate estimates, however the technique has been used successfully to estimate rates of coral reef community calcification [27,44], and is considered robust in coral reef environments when compared to other techniques [45]. The incubations of tile communities resulted in a change in A_T_ from blank-tile control chamber values that ranged from -219 to -6 μmol kg^-1^ SW in the light, and -76 to +92 μmol kg^-1^ SW in the dark. Primary A_T_ standards (CRMs; A. Dickson Laboratory, Scripps Institution of Oceanography) were titrated to calculate titrant concentration, and replicate secondary seawater standards (n = 15) were interspaced and titrated throughout incubation samples and were highly consistent (SE = 2.19 μmol kg^-1^ SW). Rates of gross photosynthesis and dark respiration (μg O_2_ cm^-2^ min^-1^), and light and dark calcification (μM CaCO_3_ cm^-2^ min^-1^), were calculated by subtracting the values of blank tile O_2_ or A_T_ values, at the end of the incubation runs, from values of chambers with tile communities, and then standardised to incubation time and planar surface area of each tile. Changes in the surface area of the tiles due to differences in settling benthos were not accounted for. Estimates of daily net photosynthesis and calcification were calculated by combining light and dark rates, assuming a square profile of 11.5 hrs light and 12.5 hrs dark. This daily estimate equated to a cumulative daily light integral of 7.45 mol photon m^-2^ d^-1^, which is similar to those at the study site and depth [42].

### Statistical analyses

Generalised linear models (GLM) were used to examine how percent cover of the different benthos OTUs on the tiles differed with median pH_T_, and across Reef (Dobu and Upa-Upasina) and Time (five vs 13 month censuses). Median pH_T_ was used in models as it was directly measured many times at each tile (rather than being calculated), and correlated better with the other carbonate chemistry parameters than A_T_ (which is not directly affected by CO_2_ addition). Percent cover data were fit using a quasibinomial distribution accounting for the larger proportion of values at the upper and lower bounds of the distribution. Non-significant (p > 0.05) main effects and interaction terms were removed in the final models. For comparison to control values (seawater pH_T_ 8.05, or Ω_Ar_ = 3.84), percent coverages of taxa were also predicted for seawater pH_T_ 7.80 or Ω_Ar_ = 2.50, using the predict function in R, as this value may be expected by the year 2100 in tropical oceans [46].

Redundancy analysis (RDA) was also conducted to visually examine how the community data separated out in two-dimensional space, and to further test the significance of each explanatory variable via permutation. The explanatory variables in the analysis included reef and census period as categorical factors, as well as the median pH_T_ at each tile as a continuous quantitative variable.

GLMs were fitted to the metabolic measurements from all tiles, using Reef and median pH_T_ of the tile origin as predictors for measurements of gross photosynthesis and respiration, and Reef and Ω_Ar_ for calcification. For the calcification assay, two chambers opened, and five outliers (with values >8-fold from the confidence intervals) were removed from final models (n = 81). A second series of GLMs was conducted on the metabolism measurements which incorporated the carbonate chemistry of the tiles, as well as the taxonomic benthic cover data (n = 66 due to the loss of 15 photos). An identity link function was used when response parameters approximated a Gaussian distribution. Quasipoisson distributions and log link functions were used for over-dispersed data. The appropriate model distribution was selected by comparing dispersion factors, preferring those which approached a value of 1. All statistical procedures were conducted with the statistical software R version 3.2.5 [47] using the packages vegan and gmodels.

## Results

### Carbonate chemistry

**Fig 1:**
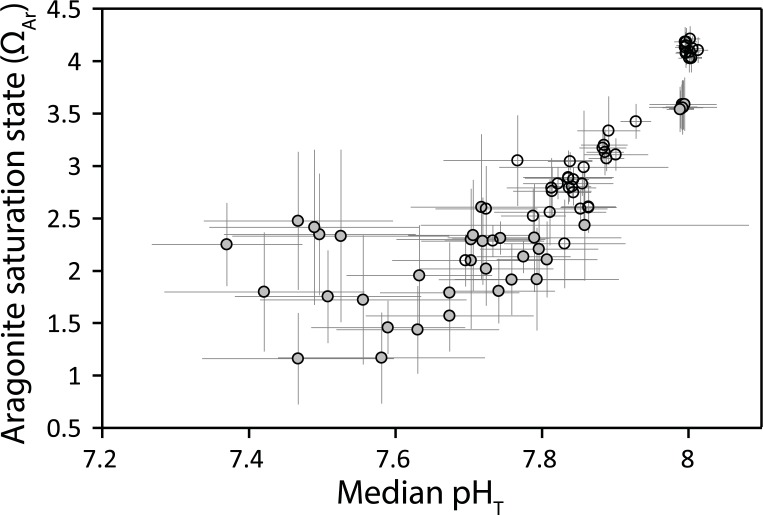
Median pH_T_ and saturation state of aragonite (Ω_Ar_) from the 88 settlement tiles. Median n = 8 pH and 5 Ω_Ar_ measures per tile. White points are from Upa-Upasina, grey are from Dobu. Error bars are standard errors.

The settlement tile communities were developed along clear CO_2_ gradients at both the Dobu and Upa-Upasina seeps (Fig 1). Both control sites had similar seawater chemistry, however the pH gradient at the Dobu seep reached lower levels than at the Upa-Upasina seep. The median seawater pH_T_ over the tiles ranged from 8.0 ± 0.001 (SE) at the control sites of both reefs, to 7.7 ± 0.10 as lowest values at the Upa-Upasina seep, and to 7.4 ± 0.10 at Dobu seep (Fig 1). Median aragonite saturation state (Ω_Ar_) was 3.84 ± 0.05 at the control sites, declining to 2.10 ± 0.25 and 1.16 ± 0.43 at the Upa-Upasina and Dobu seeps, respectively (Fig 1). Similarly, calcite saturation state (Ω_Ca_) values decreased from 5.75 ± 0.10 at the control sites along the carbonate chemistry gradient to 3.14 ± 0.38 and 1.73 ± 0.64 at the Upa-Upasina and Dobu seep, respectively. While none of the communities were exposed to median Ω_Ar_ values <1.0, 55% had median Ω_Ar_ <3.0, a value which has been suggested to be the limit for reef development [48].

Values of pCO_2_, C_T_ and A_T_ were also spread along the carbonate chemistry gradient. The daytime pCO_2_ at the control sites averaged 392.15 ± 9.32 μatm, increasing to 1008 ± 291.99 and 2253 ± 759.63 μatm at Upa-Upasina and Dobu seeps respectively. Control C_T_ values averaged 1926 ± 0.70 μmol kg^-1^, and increased to >2100 at the seeps. Median A_T_ values were slightly elevated within the seep sites (perhaps due to CaCO_3_ dissolution), and differed from control values by no more than 6%. A_T_ values increased from an average of 2252.68 ± 3.33 μmol equivalents kg^-1^ at the controls to 2339.60 ± 16.29 and 2329.68 ± 18.89 μmol equivalents kg^-1^ within the Upa-Upasina and Dobu seeps, respectively (see also S2 Fig in Enochs *et al*. [20] as their experimental units were deployed alongside the settlement tiles of the present study). Carbonate chemistry parameters were more variable within the seeps compared to the control sites (Fig 1).

### Benthic community composition

**Fig 2:**
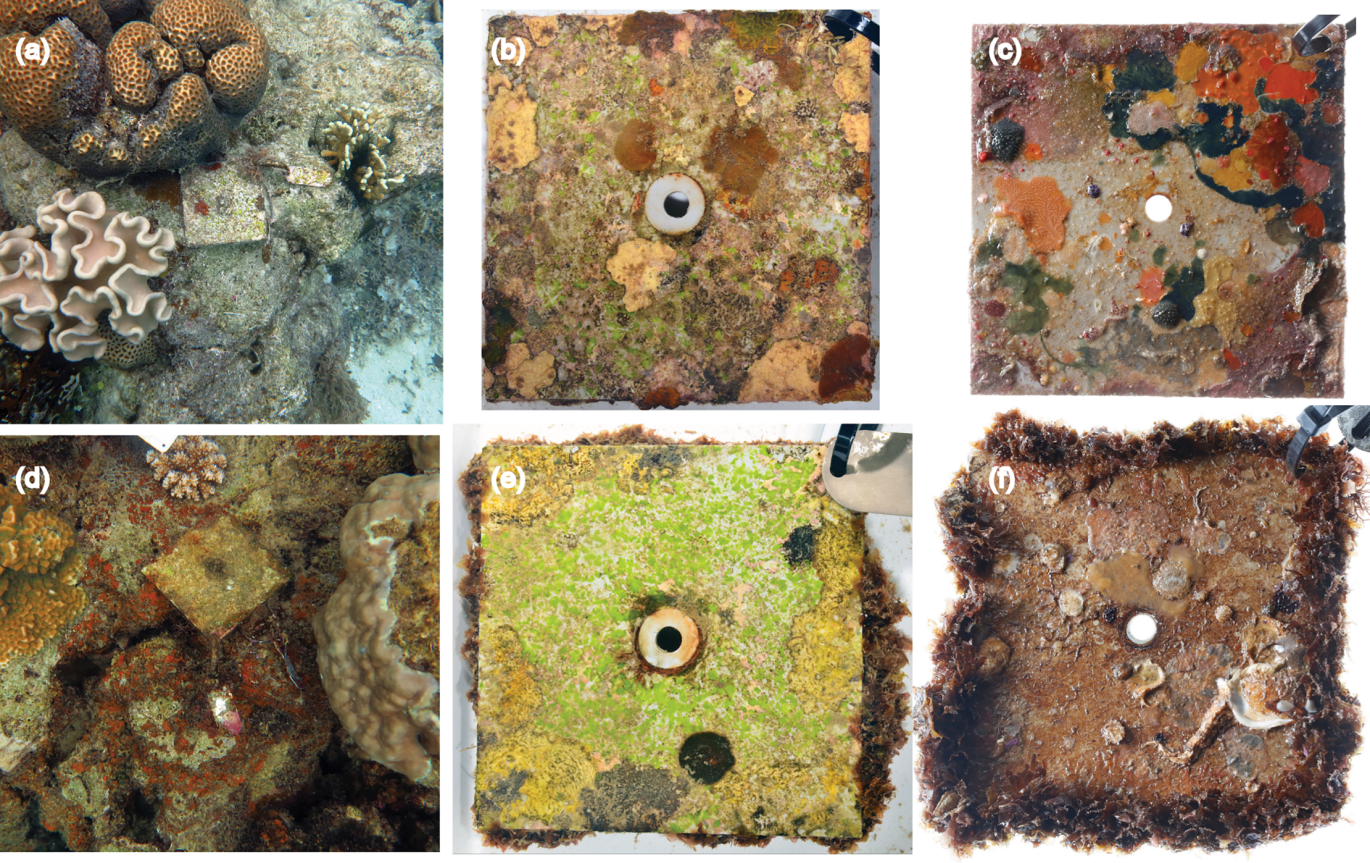
**Settlement tiles from control sites (a, b and c) and volcanic CO**_**2**_ **seep sites (d, e and f) in Papua New Guinea after 13 months deployment.** Tiles *in situ* (a and d), upper sides (b and e), and lower sides (c and f).

Both tile surfaces were covered by a significant amount of macrobenthos after five months, and after 13 months, the tiles were all but indistinguishable from the adjacent substrata (Fig 2). Communities on the upper tile sides, being exposed to higher light intensities and grazing, only included several algal OTUs and bare space, and no invertebrates. This contrasted with the lower sides of the tiles, where light and grazing intensities are low; these communities consisted of algal OTUs, as well as many invertebrate taxa and bare space (Fig 2). Coral recruits were observed on the lower tile sides [36], however, due to their small size (typically <2mm diameter), they failed to contribute to community cover estimates.

The communities underwent some successional changes between the two census periods. Pioneering algal taxa, including green filamentous algae on the upper tile side and turf algae and cyanobacteria on the lower tile side, all recorded significantly lower percent cover after 13 months compared to the five month census (GLM significant main effect of Time, all p < 0.05; Table 1). Turf algae on the upper side displayed the opposite pattern, increasing in cover between the five and 13 month censuses (S3 Table). At 13 months, several of the slower growing taxa had increased in abundance: *Peyssonnelia* spp. on the upper sides and macroalgae and sponges on the lower sides had all increased in cover, as did the combined cover of both the calcifying and the non-calcifying invertebrate groups (Table 1). The amount of unoccupied space on both the upper and lower tile sides similarly declined between the census periods as available space was progressively occupied (Table 1).

**Table 1:** Changes in percent cover of the main benthic taxonomic and functional categories, in response to pH_T_ (median per tile), Reef (contrasting Dobu against Upa-Upasina) and Time (contrasting 13 against five months of deployment).

	Estimate	SE	t	p
**Non-calcifying algae up**				
Intercept	18.88	3.13	6.04	<0.001
pH	-2.35	0.39	-5.94	<0.001
Reef	-0.45	0.13	-3.49	<0.001
**Non-calcifying algae low**				
Intercept	40.36	4.42	9.13	<0.001
pH	-5.20	0.56	-9.20	<0.001
Time	16.61	6.79	2.45	0.016
pH:Time	-2.17	0.87	-2.50	0.014
Green filaments up*				
Intercept	10.91	2.59	4.21	<0.001
pH	-1.60	0.33	-4.88	<0.001
Reef	0.79	0.13	6.15	<0.001
Time	-0.45	0.11	-4.02	<0.001
**Turf up**				
Intercept	-0.68	0.13	-5.15	<0.001
Reef	-0.93	0.17	-5.48	<0.001
Time	0.51	0.17	3.01	0.003
**Turf low**				
Intercept	-1.38	0.07	-18.43	<0.001
Time	-1.15	0.14	-7.82	<0.001
Macroalgae low*				
Intercept	26.84	4.85	5.54	<0.001
pH	-4.18	0.61	-6.83	<0.001
Reef	2.57	0.59	4.31	<0.001
Time	1.55	0.25	6.17	<0.001
Cyanobacteria low*				
Intercept	67.31	12.79	5.26	<0.001
pH	-8.79	1.63	-5.40	<0.001
Reef	-31.41	16.50	-2.33	0.021
Time	-0.32	0.17	-1.91	0.057
pH: Reef	3.99	1.72	2.32	0.022
**Peyssonnelia up**				
Intercept	14.50	4.27	3.40	<0.001
pH	-2.38	0–55	-4.33	<0.001
Time	1.74	0.26	6.65	<0.001
**Peyssonnelia low**				
Intercept	26.73	11.26	2.37	0.019
pH	-3.64	1.43	-2.54	0.012
Reef	-33.07	12.82	-2.58	0.011
Time	17.11	16.46	1.04	0.300
pH: Reef	4.15	1.63	2.54	0.012
pH: Time	-2.02	2.08	-0.97	0.334
Reef: Time	-72.00	19.88	-3.62	<0.001
pH: Reef: Time	9.04	2.52	3.59	<0.001
**CCA up**				
Intercept	-64.69	6.86	-9.43	<0.001
pH	7.89	0.86	9.13	<0.001
Reef	1.65	0.21	7.75	<0.001
Time	27.24	8.96	3.04	0.003
pH: Time	-3.37	1.13	-2.99	0.003
Reef: Time	-0.95	0.30	-3.17	0.002
**CCA low**				
Intercept	-53.013	8.45	-6.28	<0.001
pH	6.47	1.06	6.08	<0.001
Reef	-93.28	23.06	-4.04	<0.001
Time	-0.26	0.17	-1.56	0.122
pH: Reef	11.64	2.89	4.03	<0.001
Reef: Time	1.08	0.27	4.03	<0.001
Non-calcifying invertebrates low*				
Intercept	-20.90	5.71	-3.66	<0.001
pH	2.16	0.72	3.00	0.003
Reef	1.29	0.37	3.48	<0.001
Time	1.34	0.37	3.59	<0.001
Reef: Time	-1.36	0.46	-2.95	0.004
Calcifying invertebrates low*				
Intercept	-50.90	15.48	-3.29	0.001
pH	6.00	1.95	3.08	0.002
Reef	42.69	16.64	2.57	0.011
Time	1.02	0.29	3.47	<0.001
pH: Reef	-5.35	2.10	-2.55	0.012
Reef: Time	-1.03	0.41	-2.50	0.013
Ascidians low*				
Intercept	-98.37	33.45	-2.94	0.004
pH	12.00	4.20	2.86	0.005
Reef	77.74	34.19	2.27	0.024
pH: Reef	-9.65	4.29	-2.25	0.026
Polychaetes low (counts per tile)*				
Intercept	-1395.59	436.92	-3.19	0.002
pH	188.78	55.35	3.41	0.001
Reef	-39.26	17.84	-2.20	0.031
**Unoccupied space up**				
Intercept	-0.66	0.09	-7.41	<0.001
Reef	-0.62	0.14	-4.51	<0.001
Time	-1.49	0.20	-7.53	<0.001
Reef: Time	1.11	0.25	4.43	<0.001
**Unoccupied space low**				
Intercept	-13.83	4.42	-3.13	0.002
pH	1.61	0.56	2.88	0.004
Reef	-0.78	0.19	-4.04	<0.001
Time	-1.76	0.27	-6.47	<0.001
Reef: Time	0.98	0.37	2.65	0.009

Responses are for the upper (up) and lower (low) sides of the settlement tiles. Parameter estimates from the best fitting generalised linear models, with quasibinomial distributions. Non-significant terms removed from final models.

* indicates taxa only found on one tile side.

**Fig 3:**
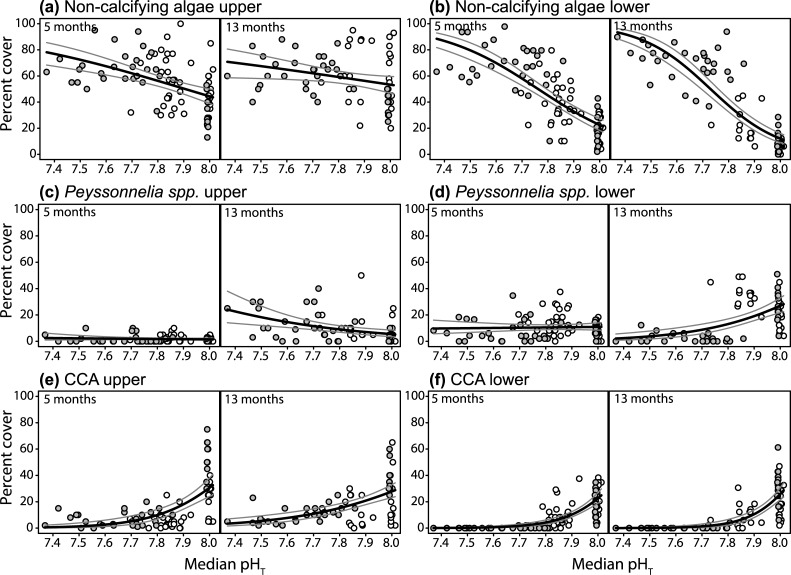
Shifts in settlement tile community composition along the pH gradients. Percent cover of non-calcifying algae on the upper (a) and lower (b) settlement tile sides, *Peyssonnelia* spp. from the upper (c) and lower (d) sides, and crustose coralline algae (CCA) from the upper (e) and lower (f) sides in relation to pH_T_. Left and right panels per plot represent the five and 13 month old communities, respectively. White points are tiles from Upa-Upasina and grey are from Dobu. The black lines represent the modelled means, while the grey lines are confidence intervals.

Changes in pH explained two and 50 times more variation in the cover of non-calcifying algae on the upper and lower tile sides, respectively, compared to the explanatory variables Reef and Time (GLM F ratios, S3 Table), indicating many patterns in the tile communities were established within five months and were largely consistent between reefs. Non-calcifying algal cover increased as pH declined from relatively low control values to ~80% at the lower end of pH gradients, without a clear threshold at which point non-calcifying algae came to dominate communities (Fig 3A and 3B). On the upper tile side the cover of non-calcifying algae increased from control values of 40.3 ± 1.7 (SE) to 56.3 ± 2.0% at pH 7.8 after five months, and 49.4 ± 2.07 to 59.2 ± 2.3% at pH_T_ 7.8 after 13 months (Fig 3), predominantly due to green filamentous algae (S1 Fig). Similarly, on the lower sides, non-calcifying algae increased from control values of 19.3 ± 1.7% to 45.1 ± 2.1% by pH 7.8 at five months, and from 8.6 ± 0.6 to 37.4 ± 2.5% by pH_T_ 7.8 at 13 months (Fig 3). Lower side communities were dominated by cyanobacteria at both seeps, and also by macroalgae at Dobu (S1 Fig).

The different taxa of calcifying algae contrasted in response to changes in pH. The very mildly calcifying red alga *Peyssonnelia* spp. on the upper surface increased in cover between census periods. In 13 month old communities, upper side *Peyssonnelia* spp. cover also increased with declining pH from control values of 4.1 ± 0.3 to 9.1 ± 1.2% at pH_T_ 7.8 (Table 1, Fig 3C and 3D). On the lower sides, *Peyssonnelia* spp. cover showed no clear patterns, indicating light limitation or competition with other benthos was co-limiting their distribution (Fig 3). The cover of the heavily calcified crustose coralline algae (CCA) declined steeply along the pH gradient on both the upper and lower tile sides (Fig 3E and 3F), and the effects of pH changes were again far stronger than Reef or Time (GLM F ratios, S3 Table). On the upper sides, CCA cover declined from control values of 35.2 ± 2.4 to 9.9 ± 1.8% by pH_T_ 7.8 in five month old communities, and from 29.6 ± 1.8 to 15.2 ± 1.5% by pH_T_ 7.8 in 13 month old communities. An apparent threshold in CCA cover was observed on the lower tile sides at pH_T_ 7.8, where CCA was virtually absent below pH_T_ 7.8, while they continued to persist with low cover on the upper sides below this pH level (Fig 3).

Patterns in the invertebrate communities were less clear compared to those seen in the algae. The cover of both calcifying and non-calcifying groups declined with pH at both reefs after five months, and at Upa-Upasina but not Dobu after 13 months (S2 Fig). Ascidian cover and the number of polychaetes significantly declined with pH at both reefs and both times, but cover differed between the reefs (Table 1). No distinct patterns were observed in the cover of bivalves, foraminifera or bryozoa (S3 Table).

**Fig 4:**
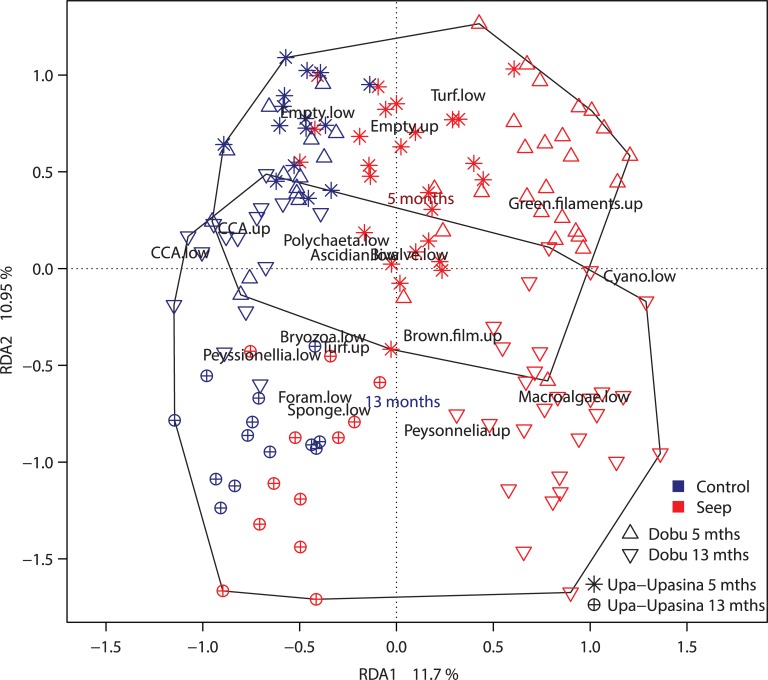
Redundancy analysis ordination of the settlement tile benthic communities at the seep and control sites. Red points represent tiles from the seeps, and blue from the controls, of Dobu and Upa-Upasina Reefs. Points in the upper polygon are five month (mths) old communities, while those in the lower polygon are at 13 months.

Results of the RDA agreed with the GLM analyses: all explanatory variables accounted for significant variation in benthic communities (ANOVA, all p = 0.001), and tile pH explained more of this variation than differences between census periods or reef (F ratios of 22.37, 19.29 and 14.67 for pH, census period and reef, respectively). In the ordination, the first RDA axis separated communities between seep and control sites. This was primarily driven by high cover of cyanobacteria, macroalgae, and green and brown filamentous algae in the high CO_2_ communities, and high CCA cover in control sites (Fig 4). Communities from Upa-Upasina clustered closer together between high CO_2_ and control tiles in comparison to Dobu, perhaps reflecting the greater intensity of CO_2_ exposure at Dobu seep. The second RDA axis divided communities between census periods: the five month old communities on the lower sides were associated with more empty space and turf algae, and on the upper sides with green filamentous algae (Fig 4). At 13 months, the communities were associated with an increased cover of *Peyssonnelia* spp., sponges, foraminifera (on the lower sides of the control tiles), and macroalgae (on the lower sides of high CO_2_ tiles).

### Benthic community metabolism

**Fig 5:**
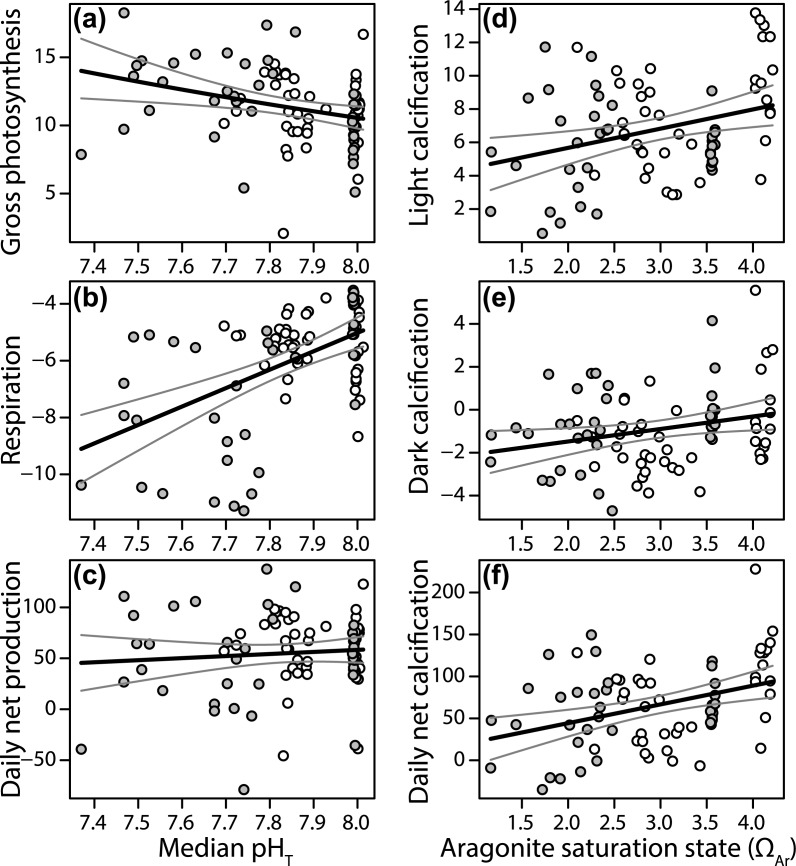
Metabolic rates from benthic communities on settlement tiles across the seawater carbon chemistry gradients. Rates of gross photosynthesis (a), respiration (b) and net daily production (c) in 13 month old communities are plotted against the median pH_T_ from the tiles. Gross photosynthesis and respiration are displayed in μg O_2_ cm^-2^ hr^-1^, and daily net production in μg O_2_ cm^-2^ day^-1^. Rates of light (d), dark (e) and net daily calcification (f) are plotted against tile median aragonite saturation state (Ω_Ar_) from the tiles. Light and dark calcification rates are displayed in μg CaCO_3_ cm^-2^ hr^-1^, and daily net calcification in μg CaCO_3_ cm^-2^ day^-1^. White points are from Upa-Upasina and grey are from Dobu. The black lines represent the modelled mean, while the grey lines are confidence intervals.

Rates of community metabolism were related to both changes in the seawater carbonate chemistry and changes in benthic communities. All metabolic measurements gradually changed along the carbonate chemistry gradient, and no abrupt changes or threshold responses were detected (Fig 5). Gross photosynthetic rates increased linearly as pH declined along the gradient (Fig 5A, Table 2). On average, control tiles produced 10.37 ± 0.4 μg O_2_ cm^-2^ hr^-1^, increasing by 10% to 11.54 ± 0.3 μg O_2_ cm^-2^ hr^-1^ at pH_T_ 7.8. Increased gross photosynthetic rates were also positively related to the increasing cover of non-calcifying algae in the upper communities, but declined with the increasing cover of non-calcifying invertebrates along the pH gradient (Table 2).

**Table 2:** Changes in community metabolism in response to median pH (gross photosynthesis; respiration) or the saturation state of aragonite (Ω_Ar_: Calcification) and Reef (contrasting Dobu against Upa-Upasina).

	Estimate	SE	t	p
**Gross photosynthesis**^**Q**^				
Intercept	6.39	1.25	1.48	0.142
pH	-1.57	0.16	-2.81	0.006
**Gross photosynthesis benthos**^**Q**^				
Intercept	-6.64	0.09	-20.21	<0.001
Non-calc invert low	-1.01	0.002	-3.78	<0.001
Non-calc algae up	1.00	0.001	3.84	<0.001
**Respiration**^**^0.25G**^				
Intercept	0.16	0.48	0.34	0.735
pH	0.05	0.06	0.80	0.427
Reef	1.75	0.53	3.29	0.001
pH: Reef	-0.22	0.07	-3.28	0.002
**Respiration benthos**^**^0.25G**^				
Intercept	1.77	0.18	9.61	<0.001
pH	-0.16	0.02	-6.69	<0.001
Bivalve	0.01	0.001	3.43	0.001
Non-calc invert low	0.001	<0.001	4.32	<0.001
**Net daily production benthos**^**G**^				
Intercept	78.10	6.48	12.04	<0.001
Bivalve	-4.97	1.70	-2.92	0.005
Non-calc invert low	-1.35	0.31	-4.37	<0.001
**Light calcification**^**G**^				
Intercept	0.06	0.02	2.76	0.007
Ω_Ar_	0.02	<0.01	2.89	0.005
**Dark calcification**^**G**^				
Intercept	-0.06	0.01	-4.27	<0.001
Ω_Ar_	0.01	<0.01	3.15	0.002
Reef	0.02	0.01	2.33	0.023
**Net daily calcification**^**G**^				
Intercept	-0.37	19.47	-0.02	0.985
Ω_Ar_	22.34	6.34	3.52	<0.001

Models were then run again but the cover of the main community members was additionally included as co-variates (benthos). Parameter estimates from the best fitting generalised linear models (^G^: Gaussian; ^Q^: quasipoisson distributions), with estimates for the quasipoisson models back-transformed to aid interpretation. Non-significant terms were removed from final models.

Respiration rates increased 20% from control site values (5.18 ± 0.6 μg O_2_ cm^-2^ hr^-1^ consumption) to those at pH_T_ 7.8 (6.21 ± 0.2), and continued to increase along the pH gradient (Fig 5B). The increase in respiration with pH was stronger at Dobu, however the pH main effect explained three times the variation in respiration compared to the interaction between pH and Reef (S4 Table, F = 33 and 10 for pH and the interaction, respectively). Respiration rates also increased with the cover of bivalves and non-calcifying invertebrates on the lower sides of the tiles (Table 2). Since declining pH elevated rates of both gross photosynthesis and respiration, no difference was detected in daily net production, which averaged 55 ± 13.2 μg O_2_ cm^-2^ day^-1^ across all tiles (Fig 5C). Daily net production was instead reduced by an increase in the cover of bivalves and non-calcifying invertebrates on the lower sides of the tiles (Table 2). Mean gross photosynthetic rates were on average twice that of respiration, and 90% of tiles recorded positive daily net production values.

Rates of community calcification in the light and dark, and net 24-h community calcification, all declined along the Ω_Ar_ gradient (Table 2). Mean light calcification rates averaged 7.73 ± 1.3 μg CaCO_3_ cm^-2^ h^-1^ at the control sites and decreased by 20% to 6.25 ± 0.4 μg CaCO_3_ cm^-2^ h^-1^ by Ω_Ar_ 2.5. None of the communities recorded net decalcification in the light, despite median Ω_Ar_ reaching as low as 1.16, while 70% of control and 80% of seeps communities showed net decalcification in the dark. Dark calcification declined from -0.01 ± 0.3 μg CaCO_3_ cm^-2^ h^-1^ at the control sites to -1.19 ± 0.2 μg CaCO_3_ cm^-2^ h^-1^ by 2.5 Ω_Ar_, and continued to decrease along the gradient (Fig 5E, Table 2). Dark calcification rates were also lower at Upa-Upasina compared to Dobu (Table 2). Daily net calcification rates of 89.14 ± 19.3 μg CaCO_3_ cm^-2^ day^-1^ at the control sites declined by 38% to 55.49 ± 5.9 at Ω_Ar_ 2.5 (equalling annual CaCO_2_ deposition of 325 vs 204 g m^-2^ yr^-1^). Given rates of light calcification were greater than those in the dark, 90% of the tiles recorded positive daily net calcification, with negative rates all observed at <2.3 Ω_Ar_ (Fig 5F). Interestingly, the declines in calcification were not significant when calcification was modelled against tile pH. Tile pH_T_ and Ω_Ar_ were highly correlated until the lower end of the pH gradient (Fig 1), where Ω_Ar_ increased relative to pH. This decoupling is likely driven by CaCO_3_ dissolution in the seeps, raising A_T_ and subsequently Ω_Ar_, and may explain the differences between model results. The inclusion of the benthic OTUs did not improve the GLM fits to the carbonate chemistry parameters for rates of light, dark or daily net calcification. No relationships were detected between gross photosynthesis and light calcification rates, or between respiration and dark calcification rates (linear regressions, all p > 0.05).

## Discussion

Ocean acidification is predicted to fundamentally alter benthic marine communities. Here we report a drastic shift in the composition and metabolism of early successional benthic coral reef communities along seawater carbonate chemistry gradients. The carbonate chemistry explained a far greater amount of change in communities than successional changes from five to 13 months, and differences between the two reefs. Shifts were more pronounced in the algae compared to the invertebrate communities, where a suite of non-calcifying algal groups largely replaced CCA on seep site tiles. Changes in CO_2_ and community composition also affected community metabolism; rates of gross photosynthesis and respiration increased with increasing CO_2_, and with the cover of certain taxonomic groups, while 24-h net calcification decreased to low or even negative values.

The present study adds to the mounting body of evidence predicting ecosystem-wide changes in benthic communities under OA. Here we found rapid increases in non-calcifying algal cover as pH declined along the carbonate chemistry gradients, and little evidence of threshold responses for these taxa. This pattern was largely consistent between light exposed and cryptic communities, with green filaments establishing dominance on the upper sides, and cyanobacteria and macroalgae on the lower sides. While results are not universal [14], patterns in benthic communities at CO_2_ seeps in the temperate Mediterranean [5,49–51], as well as multiple tropical sites in the Indo-Pacific [11,12], concur with the present study. These studies similarly predict an increase in non-calcifying algae under OA, and there are suggestions that other non-calcifying phototrophs, such as seagrasses [11] and anemones [30], may also thrive. Interestingly, non-calcifying algae have increased abundances in the wider community at the seep sites in Milne Bay [11], however they do not dominate the benthos like on the settlement tiles of the present study, or at another tropical seep [12]. Grazers are diverse and abundant at the Milne Bay seeps [52], which may prevent the proliferation of algae on upper surfaces in the wider community. Similarly, longer-term competition with benthos that had not developed fully on the tiles (e.g. the scleractinian corals) may also constrain algal growth.

Calcifying algae on the settlement tiles displayed dissimilar results between taxa along the CO_2_ gradient. The cover of the lightly calcifying algae *Peyssonnelia* spp. increased at lower pH, albeit only in high-light environments. Some *Peyssonnelia* species have increased in abundance at other seep sites [49,51], suggesting that certain species of calcifying algae may be resilient to or even benefit from OA [42,53], perhaps due to the use of aragonite over high magnesium-calcite in their skeletons [51], and by using the additional C_T_ for photosynthesis. The steep decline in CCA cover is consistent with data from multiple seep sites [8,12] and in experimentation [54] with potentially profound effects on coral reef communities [36]. The steeper decline in CCA on the lower tile surfaces, as well as the increase in *Peyssonnelia* spp. on the upper tile surfaces, indicates light intensity is playing a role in the response of these taxa to OA.

Invertebrate responses to carbonate chemistry changes varied between taxa, and overall their cover and abundances did not respond as strongly as the algae did. It is important to note that the invertebrate communities in the present study were in relatively early successional stages, and consisted of shade-adapted communities without scleractinian corals (no invertebrates were found on the upper tile surfaces). The number of tube-dwelling polychaetes per tile declined with pH, as did the cover of ascidians. Similar declines were seen in tube-dwelling polychaete species at a Mediterranean seep [55], possibly due to reduced calcification in the juvenile stage [56]. Little is known about why ascidians appear to respond negatively to elevated CO_2_, however a previous study found the abundances of ascidians on natural reef substrata also declined with CO_2_ exposure at the Milne Bay seeps [4]. Our study found no apparent effect of carbonate chemistry on the cover of the diverse groups of bivalves, bryozoans or sponges. Both bivalves and bryozoans are calcifying and considered sensitive to carbonate chemistry changes, but their CaCO_3_ skeletons are somewhat protected from the surrounding seawater through external tissue layers [10,57]. Sponges have shown a mixed response to elevated CO_2_, with some species negatively responding, while species with phototrophic symbionts or siliceous spicules may respond positively [58].

Patterns in the 13 month old tile communities were largely established in the first five months, and successional changes between census periods were considerably weaker than the influence of the carbonate chemistry gradient for the majority of taxa. While the cover of some ephemeral (turf and green filamentous algae and cyanobacteria) and slower growing taxa (*Peyssonnelia spp*., other macroalgae and the invertebrate groups) changed between census periods, this did not significantly alter the patterns in the rest of the tile communities. For example, patterns along the CO_2_ gradients in the cover of non-calcifying algae and CCA, which accounted for the majority of the tile communities, were largely consistent between census periods. This consistency between census periods contrasts a similar study at Mediterranean seeps, where Kroeker *et al*. [5] found commonalities between early settlement seep and control communities progressively diverged as competitive hierarchies were disrupted. Fabricius *et al*. [8], who closely examined patterns in CCA distributions on the tiles of the present study, concluded that it was recruitment limitation in the CCA at lower pH, rather than competition with other taxa, that established the patterns seen here.

It is unknown to what extent the successional tile communities reflect the surrounding mature benthic communities. After 13 months, the tile communities blended in and greatly resembled the surrounding benthos (personal observation). Previous work at the Milne Bay seeps has similarly shown higher turf and macroalgae cover, and lower CCA cover, on natural substrate within the seep reefs compared to control reefs [11]. Regardless of any disparities between tile and mature benthic communities, early successional communities may become increasingly prevalent on coral reefs as the frequency and severity of disturbances increases [32], making larger contributions to overall reef composition and metabolic signals, with potentially important complications for the carbonate chemistry newly settling corals will experience within the benthic boundary layer.

The present study presents the first investigation of metabolic changes for combined surface and cryptic subsurface reef communities that have developed entirely *in situ* under high CO_2_. Here we documented a 10% increase in gross photosynthesis and a 20% increase in respiration at pH_T_ 7.8 compared to control sites with a pH_T_ 8.0, but no change in net community production. Gross photosynthesis may increase under OA by directly stimulating photosynthesis [30,31], and/or by increasing the benthic cover of phototrophs [2]. Studies that have investigated metabolic changes under OA at the reef community scale are few and from quite different communities, however they have not observed the increases in gross photosynthesis reported here [17,20,27,28]. In the present study, models which included benthic community cover indicated that increases in gross photosynthesis were predominantly due to increases in the cover of non-calcareous algae, rather than the changing seawater carbonate chemistry *per se*. Respiration may increase as a consequence of increasing biomass or increased metabolism. Biomass estimates are unavailable for the tiles, however our models indicated that declining seawater pH, and increasing invertebrate cover, both significantly contributed to the observed increase in respiration at lower pH.

OA is likely reducing reef calcification rates on coral reefs [29,26]. Light, dark and net calcification rates on the tiles all declined along the Ω_Ar_ gradient, and our models indicated that it was these changes in Ω_Ar_, rather than shifts in tile communities, that were responsible. While results are not universal, OA is widely reported to reduce calcification in individual coral reef organisms [6,10] and at the community scale [17–20,29]. For example, Enochs *et al*. [20] found net daily calcification rates of light exposed coral reef communities on CaCO_3_ substrata decreased linearly along CO_2_ gradients, and became negative by pH_T_ 7.8. The stronger response found by Enochs *et al*. [20] compared to the present study is thought to be because their study used CaCO_3_ blocks as settlement substrata, and attracted many macro-boring organisms, yet did not include cryptofauna on the lower surfaces.

When predicting OA effects on coral reef calcification, one must also take the permeable carbonate matrix and sediments into consideration. These are the largest sources of reef CaCO_3_ [59], and they are more vulnerable to dissolution than calcifying organisms as they lack tissue layers to buffer them from the surrounding seawater [19,23,59,60]. For example, Comeau *et al*. [61] documented a 60% decline in the calcification of experimental coral reef communities at 1300 μatm pCO_2_, with half of this being attributed to sediment decalcification. Some estimates suggest that even if coral calcification rates are maintained under OA, the dissolution of carbonate sediments alone would result in reef loss [59,62]. Results from the calcification assays in the present study, lacking sediments and a CaCO_3_ substrata, are thus likely to considerably under-estimate reef-wide dissolution rates expected under OA. Instead, they provide insight into how the calcification dynamics of a part of the biological components of coral reef communities may respond.

There are several factors that preclude CO_2_ seep sites from perfectly representing the future of the world’s oceans. Firstly, they are relatively small, and scaling up predictions to the world’s coral reefs will undoubtedly introduce some uncertainty. Secondly, the altered carbonate chemistry at the seeps is occurring in isolation from the warming that is also predicted for a high CO_2_ world [46], and the combined effect of these two stressors can be greater than either in isolation [10]. Thirdly, the seep site A_T_ is slightly elevated (by ≤ 6% of control values), which may increase calcification rates at the seep sites [26]. Increased dissolution of carbonate sediments under OA may locally increase A_T_, however sediment dissolution- and dilution-rates from the surrounding seawater are largely unknown [59]. And finally, the seep seawater carbonate chemistry is characteristically variable over short (i.e. hourly) time-scales [63] with uncertain consequences for coral reef communities. Scleractinian corals have been shown to be largely robust, or to even benefit from increased pH variability (when compared to static lowered pH), while other coral reef organisms (e.g. CCA) can be negatively affected [64]. On the other hand, community scale studies, which allow for interactions between species and their environment, are not easily conducted in laboratory settings. While seep sites studies are not definitive, they do provide unique opportunities to overcome some issues with laboratory-based OA studies (i.e. organism acclimation and species/environment interactions) and provide further contributions to scientific consensus about the severe effects ocean acidification is afflicting on marine communities.

Results from the two seep sites investigated here, as well as other naturally occurring high CO_2_ analogues [12–14], generally agree with a plethora of experimental work from the small- [6,10,65] to large-scale [17–19,25,29,66], *in situ* seasonal comparisons [23,44] and quantitative models [67–69]. All these generally predict considerable changes for coral reefs under ‘business as usual’ carbon emissions scenarios and that we will likely see shifts in community composition, with the proliferation of non-calcifying taxa and a retraction of many calcifiers. Increases in non-calcifying algae may lead to increases in community gross production, however gross gains may be balanced by increased respiration. Ecosystem-wide calcification and CaCO_3_ accumulation rates will likely decline, owing to the carbonate chemistry changes and the dissolution of carbonate sediments and increased bio-erosion [14,20]. Unfortunately neither this study, meta-analyses [1,10], nor experimental comparisons of pre-industrial to present-day conditions [17,26] have shown signs that ecosystem acclimation will prevent the expected changes. This study further shows that many changes expected on coral reefs under increasing OA will occur along a continuum, indicating that the less CO_2_ emitted into the atmosphere, the less deviation we will see from the reefs of today.

## Supporting information

S1 Fig **Percent cover of the non-calcifying algal groups responsible for the increases seen along the pH gradient at Upa-Upasina (white points) and Dobu (grey points): green filamentous algae on the upper tile sides (a) and cyanobacteria (b) and macroalgae (c) on the lower tile sides.** Left and right hand panels represent the five and 13 month census periods, respectively. The black lines represent the modelled means, while the grey lines are confidence intervals.
(EPS)

S2 Fig **Percent cover of the sum of non-calcifying (a) and calcifying (b) invertebrates on the lower tile sides at Upa-Upasina (white points) and Dobu (grey points) in relation to median pH**_**T**_. Left and right hand panels represent the five and 13 month census periods, respectively. The black lines represent the modelled means, while the grey lines are confidence intervals.
(EPS)

S1 Table The operational taxonomic units (OTUs) and their description for the benthic groups classified on the settlement tiles. Some OTUs were further classified into more general grouping for analysis (Group).
(DOCX)

S2 Table Mean carbon chemistry parameters from the settlement tile metabolism incubation water at the two sites (Control: C and Seep: S) and two reefs Upa-Upasina (Upa) and Dobu (Dob). Standard errors are shown in brackets. N = 2 and 4 per control and seep site.
(DOCX)

S3 Table Generalised linear model results of pH, Reef (Dobu vs Upa-Upasina) and Time (five vs 13 months deployment) effects on the cover or abundance of operational taxonomic units (OTUs). Up and low refer to the upper and lower sides of the tiles, respectively. Non-significant terms removed from final models. Caption as in Table 1. *indicates taxa which were only found on one tile side.
(DOCX)

S4 Table Changes in community rates of gross photosynthesis, respiration, net daily production, and dark calcification with carbonate system parameters (median *in situ* value for each tile, numerical variable) and separately with the cover of the main OTUs (benthos) also included. Median pH was used as predictor in oxygen production and consumption models (Fig 4), while Ω_Ar_ was used in calcification models. For calcification, the inclusion of the biotic OTUs did not improve on the GLM fit to Ω_Ar_. Generalised linear model results, with ^G^ and ^Q^ denoting Gaussian and Quasi Poisson distributions used respectively, and ^**^0.25**^ indicates square root transformation. Caption as in Table 1.
(DOCX)
