# Supplementary material for: Ocean acidification alters early successional coral reef communities and their rates of community metabolism
Source: PLoS One. 2018 May 30;13(5):e0197130. doi: 10.1371/journal.pone.0197130 (PMC5976151; doi:10.1371/journal.pone.0197130)
Supplement: S1 Table — Some OTUs were further classified into more general grouping for analysis (Group). (DOCX) [file pone.0197130.s003.docx]

**S1 Table: The operational taxonomic units (OTUs) and their description for the benthic groups classified on the settlement tiles.**

| Operational  Taxonomic Unit | Description | Group |
| --- | --- | --- |
| Macro algae | Any macrophyte with a thicker, fleshy appearance, where the lamina was clearly visible with the naked eye. | Non-calcifying algae |
| Turf algae | A multi-species assemblage of filamentous algae with little to no structure observable with the naked eye. Often sediment laden. | Non-calcifying algae |
| Cyanobacteria | Filamentous cyanobacteria. | Non-calcifying algae |
| Green filamentous algae | Filamentous algae, more sparsely spaced than the turf algae group and exclusively from the phylum Chlorophyta. Only observed on the upper surface of the tiles. | Non-calcifying algae |
| Brown filamentous algae | Filamentous algae, exclusively from the phylum Phaeophyta. Only observed on the upper surface of the tiles. | Non-calcifying algae |
| Crustose coralline algae (CCA) | Non-erect, calcifying members of the order Corallinales. | Calcifying algae |
| *Peyssonnelia spp.* | Any members of the genus *Peyssonnelia.* | Calcifying algae |
| Bivalves | Any sessile members of the class Bivalvia. | Calcifying invertebrate |
| Bryozoa | Any members of the phylum Bryozoa. | Calcifying invertebrate |
| Foraminifera | Any sessile members of the phylum Foraminifera. | Calcifying invertebrate |
| Polychaeta | Any sessile, tube-forming members of the class Polychaeta | Calcifying invertebrate |
| Ascidians | Any members of the class Ascidiacea. | Non-calcifying invertebrate |
| Sponges | Any members of the phylum Porifera. | Non-calcifying invertebrate |
| Empty space | Unoccupied space where the PVC settlement tile was clearly visible. | Analysed separately |
| Unidentified | Taxa that could not be identified from the photographs. | Not analysed |

Some OTUs were further classified into more general grouping for analysis (Group).
