# Supplementary material for: Ocean acidification alters early successional coral reef communities and their rates of community metabolism
Source: PLoS One. 2018 May 30;13(5):e0197130. doi: 10.1371/journal.pone.0197130 (PMC5976151; doi:10.1371/journal.pone.0197130)
Supplement: S2 Table — Standard errors are shown in brackets. N = 2 and 4 per control and seep site. (DOCX) [file pone.0197130.s004.docx]

**S2 Table: Mean carbon chemistry parameters from the settlement tile metabolism incubation water at the two sites (Control: C and Seep: S) and two reefs Upa-Upasina (Upa) and Dobu (Dob).**

|  | Measured Parameters | | | | Calculated Parameters | | |
| --- | --- | --- | --- | --- | --- | --- | --- |
| Reef.Treatment | pH_NBS_ | Temp (°C) | A_T_ (µmol kg^-1^ SW) | C_T_ (µmol kg^-1^ SW) | *p*CO_2_ (µatm) | HCO_3_^-^ (µmol kg^-1^ SW) | Ω_Ar_ |
| Upa.C | 8.23 (0.01) | 30.5 (0.07) | 2199 (12) | 1837 (17) | 337 (12) | 1579 (19) | 4.09 (0.03) |
| Upa.S | 7.84 (0.03) | 30.0 (0.15) | 2243 (27) | 2086 (12) | 1001 (63) | 1935 (5) | 2.04 (0.15) |
| Dob.C | 8.24 (0.01) | 30.3 (0.21) | 2245 (0.1) | 1873 (5) | 334 (10) | 1606 (8) | 4.22 (0.05) |
| Dob.S | 7.84 (0.03) | 30.5 (0.25) | 2274 (9) | 2112 (19) | 1016 (84) | 1957 (24) | 2.11 (0.11) |

Standard errors are shown in brackets. N = 2 and 4 per control and seep site.
