# Supplementary material for: Ocean acidification alters early successional coral reef communities and their rates of community metabolism
Source: PLoS One. 2018 May 30;13(5):e0197130. doi: 10.1371/journal.pone.0197130 (PMC5976151; doi:10.1371/journal.pone.0197130)
Supplement: S3 Table — Up and low refer to the upper and lower sides of the tiles, respectively. Non-significant terms removed from final models. Caption as in Table 1. *indicates taxa which were only found on one tile side. (DOCX) [file pone.0197130.s005.docx]

**S3 Table: Generalised linear model results of pH, Reef (Dobu vs Upa-Upasina) and Time (five vs 13 months deployment) effects on the cover or abundance of operational taxonomic units (OTUs).**

|  | DF | Deviance | F | p |
| --- | --- | --- | --- | --- |
| **Non-calcifying algae up** | | | | |
| NULL | 151 | 25.52 |  |  |
| pH | 1 | 22.23 | 25.75 | <0.001 |
| Reef | 1 | 20.66 | 12.29 | <0.001 |
| **Non-calcifying algae low** | | | | |
| NULL | 156 | 56.67 |  |  |
| pH | 1 | 22.00 | 270.76 | <0.001 |
| Time | 1 | 21.11 | 6.95 | 0.009 |
| pH:Time | 1 | 20.30 | 6.34 | 0.013 |
| **Green filaments up*** | | | | |
| NULL | 151 | 19.65 |  |  |
| pH | 1 | 14.79 | 67.04 | <0.001 |
| Reef | 1 | 12.43 | 32.57 | <0.001 |
| Time | 1 | 11.24 | 16.43 | <0.001 |
| **Turf up** | | | | |
| NULL | 151 | 39.12 |  |  |
| Reef | 1 | 33.63 | 26.90 | <0.001 |
| Time | 1 | 31.76 | 9.15 | 0.003 |
| **Turf low** | | | | |
| NULL | 156 | 17.46 |  |  |
| Time | 1 | 12.05 | 69.84 | <0.001 |
| **Macroalgae low*** | | | | |
| NULL | 156 | 36.15 |  |  |
| pH | 1 | 22.98 | 119.09 | <0.001 |
| Reef | 1 | 18.08 | 44.32 | <0.001 |
| Time | 1 | 13.09 | 45.08 | <0.001 |
| **Cyanobacteria low*** | | | | |
| NULL | 156 | 38.47 |  |  |
| pH | 1 | 20.50 | 146.15 | <0.001 |
| Reef | 1 | 20.40 | 0.83 | 0.364 |
| Time | 1 | 19.77 | 5.12 | 0.025 |
| pH: Reef | 1 | 19.08 | 5.62 | 0.019 |
| **Brown filaments up*** | | | | |
| NULL | 151 | 13.15 |  |  |
| pH | 1 | 12.45 | 8.28 | 0.005 |
| Reef | 1 | 11.77 | 8.10 | 0.005 |
| Time | 1 | 11.36 | 4.88 | 0.029 |
| **Peyssonnelia up** |  |  |  |  |
| NULL | 149 | 16.22 |  |  |
| pH | 1 | 14.73 | 19.65 | <0.001 |
| Time | 1 | 10.33 | 57.92 | <0.001 |
| **Peyssonnelia low** | | | | |
| NULL | 156 | 22.00 |  |  |
| pH | 1 | 19.24 | 26.94 | <0.001 |
| Reef | 1 | 18.39 | 11.08 | 0.001 |
| Time | 1 | 16.65 | 22.70 | <0.001 |
| pH : Reef | 1 | 13.36 | 42.83 | <0.001 |
| pH : Time | 1 | 11.79 | 20.42 | <0.001 |
| Reef : Time | 1 | 11.38 | 5.35 | 0.022 |
| pH : Reef : Time | 1 | 10.37 | 13.14 | <0.001 |
| **CCA up** | | | | |
| NULL | 151 | 31.23 |  |  |
| pH | 1 | 20.49 | 112.75 | <0.001 |
| Reef | 1 | 13.76 | 70.63 | <0.001 |
| Time | 1 | 13.75 | 0.02 | 0.884 |
| pH: Time | 1 | 13.22 | 5.56 | 0.020 |
| Reef: Time | 1 | 12.26 | 10.06 | 0.002 |
| **CCA low** | | | | |
| NULL | 156 | 28.65 |  |  |
| pH | 1 | 11.31 | 310.40 | <0.001 |
| Reef | 1 | 11.31 | 0.0004 | 0.984 |
| Time | 1 | 11.27 | 0.79 | 0.374 |
| pH: Reef | 1 | 9.48 | 32.05 | <0.001 |
| Reef: Time | 1 | 8.54 | 16.73 | <0.001 |
| **Non-calcifying invertebrates low*** | | | | |
| NULL | 151 | 13.41 |  |  |
| pH | 1 | 12.81 | 7.05 | 0.009 |
| Reef | 1 | 12.29 | 6.19 | 0.014 |
| Time | 1 | 11.84 | 5.21 | 0.023 |
| Reef: Time | 1 | 11.06 | 9.13 | 0.003 |
| **Calcifying invertebrates low*** | | | | |
| NULL | 151 | 10.50 |  |  |
| pH | 1 | 9.86 | 9.05 | 0.003 |
| Reef | 1 | 9.81 | 0.61 | 0.437 |
| Time | 1 | 9.20 | 8.56 | 0.004 |
| pH: Reef | 1 | 8.57 | 8.86 | 0.003 |
| Reef: Time | 1 | 8.12 | 6.32 | 0.013 |
| **Ascidians low*** | | | | |
| NULL | 156 | 28.76 |  |  |
| pH | 1 | 27.73 | 5.69 | 0.018 |
| Reef | 1 | 25.25 | 16.62 | <0.001 |
| pH: Reef | 1 | 24.04 | 6.63 | 0.011 |
| **Polychaeta low*** | | | | |
| NULL | 156 | 7.43 |  |  |
| pH | 1 | 7.09 | 5.28 | 0.023 |
| Reef | 1 | 6.39 | 10.78 | 0.001 |
| **Polychaeta low (counts per tile)*** | | | | |
| NULL | 85 | 638166 |  |  |
| pH | 1 | 514835 | 21.04 | <0.001 |
| Reef | 1 | 486462 | 4.84 | 0.031 |
| **Bryozoa low*** | | | | |
| NULL | 156 | 25.99 |  |  |
| pH | 1 | 24.45 | 8.42 | 0.004 |
| Reef | 1 | 24.45 | 0.03 | 0.860 |
| Time | 1 | 21.74 | 14.77 | <0.001 |
| pH : Reef | 1 | 18.94 | 15.35 | <0.001 |
| Reef : Time | 1 | 17.62 | 7.17 | 0.008 |
| **Sponge low*** | | | | |
| NULL | 156 | 12.21 |  |  |
| Time | 1 | 9.40 | 31.57 | <0.001 |
| **Foraminifera low*** | | | | |
| NULL | 156 | 2.08 |  |  |
| pH | 1 | 1.94 | 15.28 | <0.001 |
| Reef | 1 | 1.65 | 32.62 | <0.001 |
| Time | 1 | 0.84 | 90.21 | <0.001 |
| **Unoccupied space up** | | | | |
| NULL | 151 | 18.59 |  |  |
| Reef | 1 | 17.74 | 11.24 | 0.001 |
| Time | 1 | 13.61 | 54.65 | <0.001 |
| Reef: Time | 1 | 12.04 | 20.73 | <0.001 |
| **Unoccupied space low** | | | | |
| NULL | 156 | 23.93 |  |  |
| pH | 1 | 22.26 | 17.51 | <0.001 |
| Reef | 1 | 20.56 | 17.81 | <0.001 |
| Time | 1 | 14.91 | 59.13 | <0.001 |
| Reef: Time | 1 | 14.22 | 7.16 | 0.008 |

Up and low refer to the upper and lower sides of the tiles, respectively. Non-significant terms removed from final models. Caption as in Table 1. *indicates taxa which were only found on one tile side.
