# Supplementary material for: Ocean acidification alters early successional coral reef communities and their rates of community metabolism
Source: PLoS One. 2018 May 30;13(5):e0197130. doi: 10.1371/journal.pone.0197130 (PMC5976151; doi:10.1371/journal.pone.0197130)
Supplement: S4 Table — Median pH was used as predictor in oxygen production and consumption models (Fig 4), while ΩAr was used in calcification models. For calcification, the inclusion of the biotic OTUs did not improve on the GLM fit to ΩAr. Generalised linear model results, with G and Q denoting Gaussian and Quasi Poisson distributions used respectively, and ^0.25 indicates square root transformation. Caption as in Table 1. (DOCX) [file pone.0197130.s006.docx]

**S4 Table: Changes in community rates of gross photosynthesis, respiration, net daily production, and dark calcification with carbonate system parameters (median *in situ* value for each tile, numerical variable) and separately with the cover of the main OTUs (benthos) also included.**

|  | DF | Deviance | | F | p | |  |  |
| --- | --- | --- | --- | --- | --- | --- | --- | --- |
| **Gross photosynthesis^Q^** | | | | | | |  |  |
| NULL | 80 | 1.03 | |  |  | |  |  |
| pH | 1 | 0.94 | | 7.71 | 0.007 | |  |  |
| **Gross photosynthesis benthos^Q^** | | | | |  | |  |  |
| NULL | 65 | 0.77 | |  |  | |  |  |
| Non-calc invert low | 1 | 0.65 | | 14.80 | <0.001 | |  |  |
| Non-calc algae up | 1 | 0.52 | | 15.11 | <0.001 | |  |  |
| **Respiration^^0.25G^** | | | | | | |  |  |
| NULL | 80 | 0.15 | |  |  | |  |  |
| pH | 1 | 0.11 | | 33.24 | <0.001 | |  |  |
| Reef | 1 | 0.11 | | 0.73 | 0.396 | |  |  |
| pH: Reef | 1 | 0.10 | | 10.77 | 0.001 | |  |  |
| **Respiration benthos^^0.25G^** | | | | | | |  |  |
| NULL | 63 | 0.14 | |  |  | |  |  |
| pH | 1 | 0.10 | | 38.06 | <0.001 | |  |  |
| Bivalve | 1 | 0.08 | | 11.40 | 0.001 | |  |  |
| Non-calc invert low | 1 | 0.06 | | 18.66 | <0.001 | |  |  |
| **Net daily production benthos^G^** | | | | | | |  |  |
| NULL | 63 | 1.05e^5^ | |  |  | |  |  |
| Bivalve | 1 | 9.51e^4^ | | 7.96 | 0.006 | |  |  |
| Non-calc invert low | 1 | 7.24e^4^ | | 19.08 | <0.001 | |  |  |
| **Light calcification^G^** | | | | | | |  |  |
| NULL | 77 | 0.19 | |  |  | |  |  |
| Ω_Ar_ | 1 | 0.18 | | 8.37 | 0.005 | |  |  |
| **Dark calcification^G^** | | | | | | |  |  |
| NULL | 79 | 0.07 | |  |  | |  |  |
| Ω_Ar_ | 1 | 0.07 | | 5.88 | 0.018 | |  |  |
| Reef | 1 | 0.0.06 | | 5.40 | 0.023 | |  |  |
| **Net daily calcification^G^** | | | | | | |  |  |
| NULL | 76 | 1.79e^5^ | |  |  | |  |  |
| Ω_Ar_ | 1 | 1.53e^5^ | | 12.43 | <0.001 | |  |  |
|  |  | |  | | |  | |  |

Median pH was used as predictor in oxygen production and consumption models (Fig 4), while Ω_Ar_ was used in calcification models. For calcification, the inclusion of the biotic OTUs did not improve on the GLM fit to Ω_Ar_. Generalised linear model results, with ^G^ and ^Q^ denoting Gaussian and Quasi Poisson distributions used respectively, and **^^0.25^** indicates square root transformation**.** Caption as in Table 1.
